# Anaphylaxis to African finger millet with exercise as a possible co-factor

**DOI:** 10.1186/2045-7022-5-S3-P139

**Published:** 2015-03-30

**Authors:** Sandhya Limaye, Jocelyn Jiang, Martina Rafferty

**Affiliations:** 1 Concord Hospital, Sydney, Australia

## 

The following case report describes the rare occurrence of anaphylaxis to African finger millet with exercise as a possible co-factor. An young adult with no pets nor background history of atopic disease, consumed for the first time a Sri Lankan pancake made from “kurakkan flour”, coconut and sugar. The patient then undertook a strenuous walk, following which he developed anaphylaxis requiring emergency treatment with Adrenaline, nebulised Salbutamol, corticosteroids and antihistamines.

On subsequent review, the patient reported no further reactions and had reintroduced coconut into their diet. They had not ingested any further “kurakkan flour”, but continued with a regular and intensive exercise regimen with no clinical reactions. Skin prick tests demonstrated sensitisation to dustmite and grass pollens; common food allergens tested were all negative. A preparation of the implicated flour mixed with saline into a thin paste was used for skin testing and the patient demonstrated a 6 x 6mm positive reaction. Three healthy volunteers tested negative to the same preparation.

Kurakkan flour is prepared from African finger millet and is commonly used in Sri Lankan cooking. Anaphylaxis to millet was first described in 1981,[1] however a limited number of case reports suggests this is a rare reaction. More recent publications describe hypersensitivity in bird keepers with sensitisation occurring via inhalational exposure to millet in birdseed.[2] Millet sensitisation has been demonstrated in bird keepers both with and without a history of clinical reactivity.[3] Cross reactivity between rice and millet species can occur secondary to sensitisation to a 16kD allergen in rice grains.[4] Notably, the major prolamin in millet is panicin, which has not been shown to demonstrate cross-reactivity with omega-5-gliadin, the allergenic wheat prolamin implicated in wheat-dependent exercise-induced anaphylaxis.

Although cases of millet anaphylaxis have been described with no prior history of sensitisation,[5,6] this is the first report describing millet anaphylaxis with exercise as a possible co-factor. Whilst millet is not a commonly used food, the growing popularity of gluten-free and “natural” diets may result in an increasing frequency of clinical reactivity in Western populations.
